# Analyzing the molecular mechanism of Scutellaria Radix in the treatment of sepsis using RNA sequencing

**DOI:** 10.1186/s12879-024-09589-2

**Published:** 2024-07-12

**Authors:** Yaxing Deng, Linghan Leng, Chenglin Wang, Qingqiang Yang, Yingchun Hu

**Affiliations:** 1 Department of General Surgery (Gastrointestinal Surgery), The Affiliated Hospital of Southwest Medical University, 25 Taiping Street, Jiangyang District, Luzhou, Sichuan China; 2 Department of Emergency Medicine, The Affiliated Hospital of Southwest Medical University, 25 Taiping Street, Jiangyang District, Luzhou, Sichuan China

**Keywords:** Traditional Chinese medicine, Scutellariae Radix, Sepsis, RNA sequencing, Network pharmacology, Molecular docking

## Abstract

**Background:**

Sepsis is a life-threatening organ dysfunction, which seriously threatens human health. The clinical and experimental results have confirmed that Traditional Chinese medicine (TCM), such as Scutellariae Radix, has anti-inflammatory effects. This provides a new idea for the treatment of sepsis. This study systematically analyzed the mechanism of Scutellariae Radix treatment in sepsis based on network pharmacology, RNA sequencing and molecular docking.

**Methods:**

Gene expression analysis was performed using Bulk RNA sequencing on sepsis patients and healthy volunteers. After quality control of the results, the differentially expressed genes (DEGs) were analyzed. The active ingredients and targets of Scutellariae Radix were identified using The Traditional Chinese Medicine Systems Pharmacology Database and Analysis Platform (TCMSP). Gene Ontology (GO) and Protein-Protein Interaction (PPI) analysis were performed for disease-drug intersection targets. With the help of GEO database, Survival analysis and Meta-analysis was performed on the cross-targets to evaluate the prognostic value and screen the core targets. Subsequently, single-cell RNA sequencing was used to determine where the core targets are located within the cell. Finally, in this study, molecular docking experiments were performed to further clarify the interrelationship between the active components of Scutellariae Radix and the corresponding targets.

**Results:**

There were 72 active ingredients of Scutellariae Radix, and 50 common targets of drug and disease. GO and PPI analysis showed that the intersection targets were mainly involved in response to chemical stress, response to oxygen levels, response to drug, regulation of immune system process. Survival analysis showed that PRKCD, EGLN1 and CFLAR were positively correlated with sepsis prognosis. Meta-analysis found that the three genes were highly expressed in sepsis survivor, while lowly in non-survivor. PRKCD was mostly found in Macrophages, while EGLN1 and CFLAR were widely expressed in immune cells. The active ingredient Apigenin regulates CFLAR expression, Baicalein regulates EGLN1 expression, and Wogonin regulates PRKCD expression. Molecular docking studies confrmed that the three active components of astragalus have good binding activities with their corresponding targets.

**Conclusions:**

Apigenin, Baicalein and Wogonin, important active components of Scutellaria Radix, produce anti-sepsis effects by regulating the expression of their targets CFLAR, EGLN1 and PRKCD.

## Background

Sepsis is a complex clinical syndrome, defined as a life-threatening organ dysfunction caused by host response to infection [1], which is characterized by unbalanced pro-inflammatory and anti-inflammatory response and progressive organ damage, including systemic inflammatory response syndrome (SIRS), acute respiratory distress syndrome (ARDS), acute lung injury (ALI), and liver, kidney, brain and other multiple organ dysfunction syndrome (MODS). The clinical symptoms of sepsis can be persistent hypotension, progressive metabolic acidosis, diffuse blood coagulation dysfunction, etc [2, 3]. Although many studies are devoted to the specific treatment of sepsis, non-specific supportive therapy, such as antibiotics and fluid resuscitation, remains the only treatment of sepsis, due to the complexity of its pathological mechanisms as well as the diversity of clinical manifestations [4]. Worldwide, sepsis with high mortality poses a great threat to human life and a significant economic burden, necessitating the emergence of specific drugs to effectively reverse sepsis. The poor prognosis of sepsis is closely related to the body metabolism and immune dysfunction, and the key to treating sepsis lies in effectively addressing the metabolic and immune dysregulation in patients with sepsis [1]. In recent years, TCM has become widely recognized for its ability to regulate the body’s metabolism and immune system, and also shows advantages in anti-infection, antiviral and anti-tumor aspects.

Sepsis treatment is complex, and a single chemical drug may be difficult to become specific drugs for sepsis. Conversely, since TCMs contains multiple active ingredients with multiple pharmacological effects, this creates unique opportunities for the treatment of sepsis [5]. Scutellariae Radix is a complex of various active ingredients, which has the effects of heat-clearing and fire-purging, detoxifying, stopping bleeding, preventing abortion. Previous studies have found that its active components such as Wogonin, Baicalein, Baicalin, and Apigenin have the biological effects of antibacterial, anti-inflammatory, anti-oxidation, and anti-tumor, and have been widely used in hepatitis, pneumonia, jaundice, dysentery, diarrhea, and tumor [6].

**Fig. 1:**
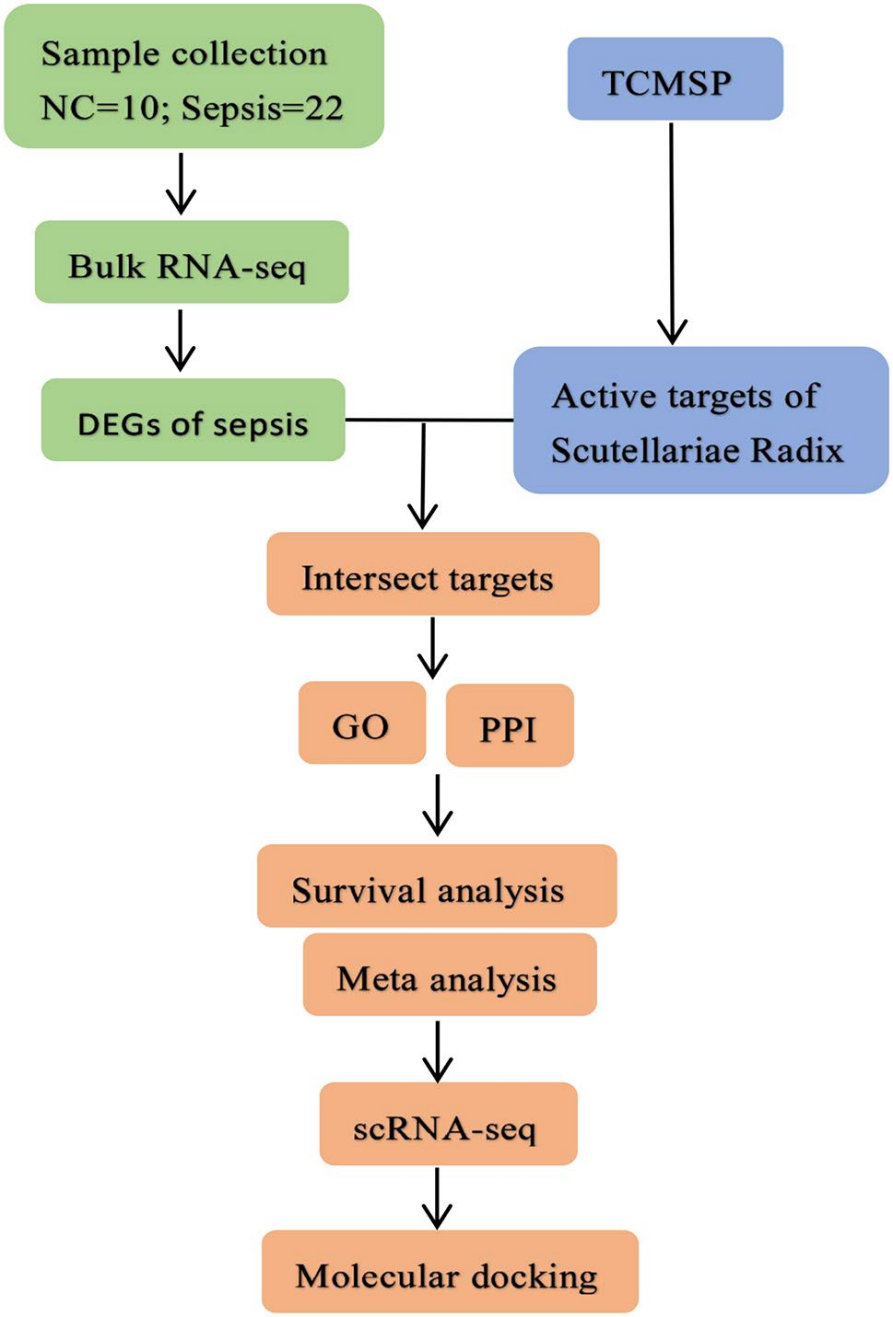
Flow chart. The peripheral blood of 22 septic patients and 10 healthy volunteers was collected for Bulk RNA sequencing and differential expression analysis. At the same time, the TCMSP database was used to obtain the active ingredients and targets of Scutellariae Radix. Disease-drug intersection targets are used for GO and PPI analysis. Core targets were screened using survival analysis and meta-analysis, then single-cell RNA sequencing was performed on core targets to clarify cell line localization. Subsequently, the potential active ingredients and targets of Scutellariae Radix in the treatment of sepsis were screened. Finally, molecular docking experiments were performed to further verify the interrelationship between the drug active ingredient and the target

Plant drugs have been used in health care and disease treatment for thousands of years, but the composition of TCM is complex, and the mechanism of action still lacks in-depth research from clinical, individual, cell and gene regulation. Network pharmacology can be systematically analyzed through complex network models to explore the interaction relationship between TCMs active ingredients-target-disease, so as to establish a foundation for researching the action mechanisms of different TCMs active ingredients and their contemporary medical uses [7]. Simultaneously, with the rapid advancement of RNA sequencing technology, we are able to gain a deeper genetic insight into sepsis. Therefore, this study used network pharmacology and RNA sequencing technology to provide a more objective interpretation of the molecular mechanism related to the active ingredients of Scutellariae Radix for sepsis, in order to provide possible targets and lead compounds for new drug development in sepsis. The specific process is shown in Fig. 1.

## Methods

### Candidate recruitment

Peripheral blood samples from healthy volunteers (*n* = 10) and sepsis patients (*n* = 22) in the Affiliated Hospital of Southwest Medical University from January 2019 to December 2020 were collected. There were four inclusion criteria: (1) Patients with sepsis hospitalized by Emergency Intensive Care Units (EICUs); (2) Meeting the Sepsis 3.0 guideline published in 2016; (3) The age of the patient ≥ 16 years and ≤ 95 years; (4) Informed consent was signed by the patient or his/her legal representative. Exclude patients with prior organ failure, prior immune system disorders, and prior hematological disorders. Clinical information and inflammatory indicators of the included subjects were collected, including the Age, AST, ALT, DBIL, TBIL, creatinine, urea, uric acid, total leukocyte count, neutrophil count, monocyte count, and lymphocyte count. The above-mentioned data of inflammatory indicators were derived from the auxiliary tests of blood routine, liver function and renal function of these patients. Data analysis was performed using Graphpad Pism9.5, the unpaired t-test was selected, and the mean ± standard deviation was calculated for each item. Ethics Committee approval of the Affiliated Hospital of Southwest Medical University has been obtained for this study, which complies with the Declaration of Helsinki and medical ethics standards (ethics number: ky2018029), and the clinical trial registration number: ChiCTR1900021261.

### Bulk RNA sequencing

To obtain the RNA sequences of the included subjects, we performed the Bulk RNA Sequencing. After centrifuging the blood samples for 5 min at 12,000 rpm at 4℃, the supernatant was transferred to 0.3 mL chloroform in an EP tube. At 4℃, the mix was centrifuged at 12,000 rpm for 10 min. The upper aqueous phase after centrifugation contained the RNA that we needed. Mix this aqueous phase with an equal volume of supernatant of isoprol alcohol in a new tube, and the mix was centrifuged at 13,600 rpm at 4℃ for 20 min. With 1 mL 75% ethanol, the pellet was washed twice after deserting the supernatant. The RNA was then dissolved in 100 µL of DEPC-treated water. mRNA was purified using oligo(dT)-attached magnetic beads. Purified mRNA was fragmented into small pieces with fragment buffer at appropriate temperature. After first-strand cDNA was generated using random hexamer-primed reverse transcription, second-strand cDNA was synthesized. The cDNA fragments obtained from previous step were amplified by PCR, and products were purified by Ampure XP Beads, then dissolved in EB solution. Sequencing reactions were performed by the PacBio Sequel sequencer (BGI-Shenzhen, China) with Sequel Sequencing Kit 2.1 and Sequel SMRT Cell 1 M v2 Tray. The clean reads were mapped to the reference genome (Genome Reference Consortium Human Build 38) using HISAT2 (v2.0.4) [8]. Bowtie2 (v2.2.5) [9] was applied to align the clean reads to the reference coding gene set, then expression level of gene was calculated by RSEM (v1.2.12) [10]. RNA sequencing datasets for this study are available in the China National GeneBank DataBase (CNGBdb) at https://db.cngb.org/. Accession codes: CNP0002611.

### Differentially expressed RNA analysis

Differential gene expression is a prerequisite for the analysis of gene function. We performed differential analysis of the RNA sequencing data to expect the differences between sepsis patients and healthy volunteers at the gene level, which may be the key to the treatment of sepsis. In order to identify outliers and screen out clusters of samples with high similarity, we performed Principal Component Analysis (PCA) on sample data using the online platform iDEP 1.0 (http://149.165.154.220/idepg/) [11]. Subsequently, the distribution density and box plot analysis of the sample data after quality control and filtering were performed to clarify the homogeneity and comparability of the data. The DESeq2 method was used for statistical analysis, and the absolute value of fold change (FC) ≥ 2 and false discovery rate (FDR) < 0.05 were used as the criteria to screen for differentially expressed genes.

### Scutellariae Radix active ingredients and targets screening

Various active components and targets of Scutellariae Radix have different mechanisms of action and therapeutic effects, and screening out specific active ingredients is the key to understand the mechanism of Scutellaria baicalensis in treating sepsis. TCMSP (http://tcmspw.com/tcmsp.php) is widely used for pharmacological analysis for TCMs to obtain the relationship between drugs, active ingredients, targets and diseases [12]. The active ingredients and targets of Scutellariae Radix were screened by TCMSP, the drug-likeness (DL) ≥ 0.18 was set as the standard. Then the active ingredients were obtained and the corresponding target was collected.

### Scutellariae Radix ingredient-target network construction

In order to further clarify the target of Scutellariae Radix in the treatment of sepsis and elucidate its therapeutic mechanism, the intersection of active target of Scutellariae Radix and the differentially expressed gene of sepsis are collected, the intersection is the potential target for anti-sepsis of Scutellariae Radix. For further clarification of its mechanism in the treatment of sepsis, Cytoscape 3.8.2 was used to construct the active ingredient-intersection target network of Scutellariae Radix.

### GO analysis

The GO functional enrichment analysis, which includes Biological Processes (BP), Cellular Components (CC) and Molecular Functions (MF), is a typical method of analyzing gene data. In order to view the functional enrichment of the differential expressed genes globally and understand the functions and characteristics, GO analysis was performed on the intersecting genes using R4.0.5, the first 20 gene sets of BP were enriched, and *P* < 0.05 was defined as statistically significant.

### PPI analysis

The PPI network is based on the strength of the interactions between two proteins. It is theoretically more likely that a protein will be a core target if it is closer to the middle region and has more connections to external factors. There is a commonly used platform for analyzing protein interactions called STRING Database (https://string-db.org/). For further screening of the core genes, we submit the collected Scutellariae Radix active target with the sepsis differential expression intersection gene to the STRING database, select “Homo sapiens” in the species option, set the correlation intensity coefficient to 0.3, and remove points in the network that are not connected.

### Survival analysis

Clinical data is the basis of scientific research, in order to better explore the relationship between genes and prognosis and reducing the range of the core genes, public dataset GSE65682 [13] from the GEO database (https://www.ncbi.nlm.nih.gov/geo/) was used to further clarify the relationship between the active targets of Scutellariae Radix and the prognosis of sepsis. GSE65682 consists of 479 peripheral blood samples from patients with sepsis, including gene expression values and clinical prognostic data for each patient. Graphpad prism7 was used for survival analysis, the significance of a logrank test is defined as a P-value of 0.05.

### Meta-analysis

To more accurately evaluate the expression of core genes in different groups and clarify whether the expression differences of core genes in different groups are statistically significant, we downloaded the sepsis datasets GSE54514 [14], GSE63042 [15], GSE95233 [16] from the GEO database. Data were divided into sepsis survivor and non-survivor group, and meta-analysis was performed on single genes in different datasets based on R packs.

### Single-cell RNA sequencing

Peripheral blood cells are a mixture of multicellular lines, single-cell RNA sequencing can help researchers to conduct cellular localization analysis of target genes, thus identifying appropriate cell lines for subsequent molecular mechanism studies. We collected five peripolar blood samples for high-throughput sequencing. The raw reads generated by sequencing is a fastq format, and the raw reads is analyzed and compared to the reference genome (Genome Reference Consortium Human Build 38) by using 10× genomics official software CellRanger. Based on the preliminary quality control of Cellranger, the data was further analyzed and processed using the Seurat software package [17]. Specifically, the FindAllMarkers function in the Seurat package [18] was used to identify the marker genes, that is, to find genes that were upregulated differently in each cell line relative to other cell populations, and these genes were potential marker genes for each cell line. Using the VlnPlot and FeaturePlot functions, a marker gene was visualized. To calculate the correlation between the expression profiles of the cells and the reference dataset, cell types with the highest correlation in the reference dataset were assigned to the cells to be identified using the SingleR package [19]. As a result, a single-cell RNA bank related to sepsis will be established.

### Molecular docking

Molecular docking refers to the process of mutual recognition between two or more molecules in three-dimensional space through geometric matching and energy matching. By finding the most stable interaction mode between drug and receptor, the binding free energy of the interaction between the two can be measured, and thus the therapeutic effect of the drug can be predicted. In order to further verify the molecular mechanism of Scutellariae Radix in the treatment of sepsis, the main active ingredients of Scutellariae Radix were downloaded from Pubchem database and retained as SDF format data. The X-ray crystal structures of the core target was obtained from the PDB database (http://www.rcsb.org/) [20], and optimized treatment of the target was carried out using PyMol-2.1.0 software (https://pymol.org/2/) [21], including eliminate water molecules and pro-ligand small molecules. Meanwhile, hydrogenation and charge treatment were carried out on the target and the resulting output is in pdbqt format with AutoDock Tools-1.5.6. With the drug as the ligand and the corresponding target as the receptor, the molecular docking experiments were performed via vina-2.0 inside pyrx software and the binding energy was calculated. The best affinity conformation was chosen as the final docking conformation. By using PyMol software (https://pymol.org/2/), the results were visualized as 3D diagrams. Further, Discovery Studio 2020 Client (https://discover.3ds.com/discovery-studio-visualizer-download) was used to draw 2D diagrams.

## Results

### Clinical characteristics of samples

A total of 22 cases of sepsis and 10 cases of normal control were included in this study. The mean age of sepsis group was 56.09 years, while the control group was 53.5 years. AST, ALT, DBIL, TBIL, creatinine, urea, uric acid, total leukocyte, neutrophil, monocyte and lymphocyte were statistically analyzed using the unpaired t-test. Then, the means and standard deviations were calculated (Table 1). Compared with healthy volunteers, the inflammatory indicators of patients with sepsis were significantly increased, such as neutrophil (*P* < 0.05).

**Table 1:** Clinical information of septic patients and normal controls

Item	Sepsis(*n*=22)	NC (*n*=10)	*P* value
age (years)	56.09±3.733	53.5±2.423	0.6590
AST(U/L)	146.8±59.24	22.18±1.431	0.1703
ALT(U/L)	90.02±38.9	20.94±2.037	0.2449
DBIL(μmol/L)	16.74±3.152	5.24±0.6478	0.0214
TBIL(μmol/L)	32.3±8.203	16.79±2.014	0.2187
creatinine (μmol/L)	106.5±23.27	63.75±2.928	0.2299
urea (mmol/L)	10.74±2.116	5.046±0.4708	0.0838
uric acid(μmol/L)	288.3±36.9	371.4±19.83	0.1533
total leukocyte (10^9/L)	12.88±1.528	6.877±0.5844	0.0147
neutrophil(10^9/L)	13.67±2.83	4.128±0.364	0.0319
monocyte(10^9/L)	0.8086±0.2311	0.443±0.05793	0.3019
lymphocyte(10^9/L)	1.145±0.3337	2.02±0.1806	0.0985

The Age, AST, ALT, DBIL, TBIL, creatinine, urea, uric acid, total leukocyte count, neutrophil count, monocyte count, lymphocyte count of 22 sepsis and 10 normal controls, data were expressed as mean ± standard deviation

### Differentially expressed RNA analysis

**Fig. 2:**
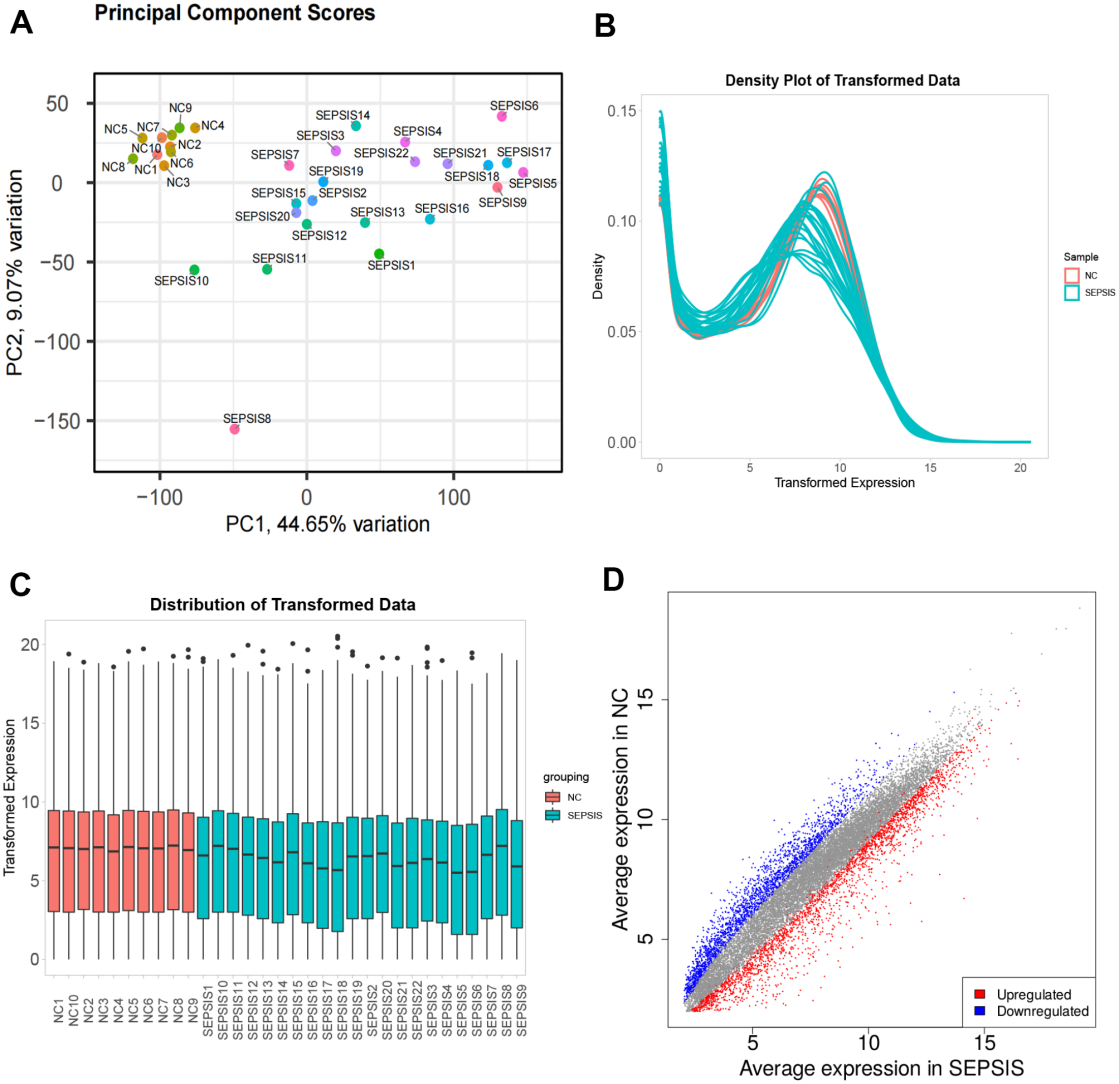
Data quality control and differential RNA screening. (**A**), PCA analysis showed that the two groups of samples could be clearly distinguished, and there were no outlier samples. (**B**-**C**) Density distribution and box plot showed that the data of each sample were homogeneous and comparable. (**D**), Volcano map shows 2462 up-regulated differentially expressed genes in red, 2046 down-regulated differentially expressed genes in blue, the abscissa is the average expression of genes in sepsis samples, and the ordinate is the average expression of genes in normal samples

PCA analysis of the sequenced mRNA showed that the distinction between normal samples and sepsis samples was good, no outlier samples were found (Fig. 2A). The density distribution and box plot showed that the data of two groups were homogeneous and comparable (Fig. 2B-C). The data from the two sets were compared using the difference analysis, and the absolute value of FC ≥ 2 and FDR < 0.05 were used as the standards, then 4508 differentially expressed RNAs were screened, of which red indicated 2462 upregulated RNAs, blue represented 2046 downregulated RNAs, and gray showed no difference RNAs (Fig. 2D).

### Scutellariae radix active ingredients and targets screening

**Fig. 3:**
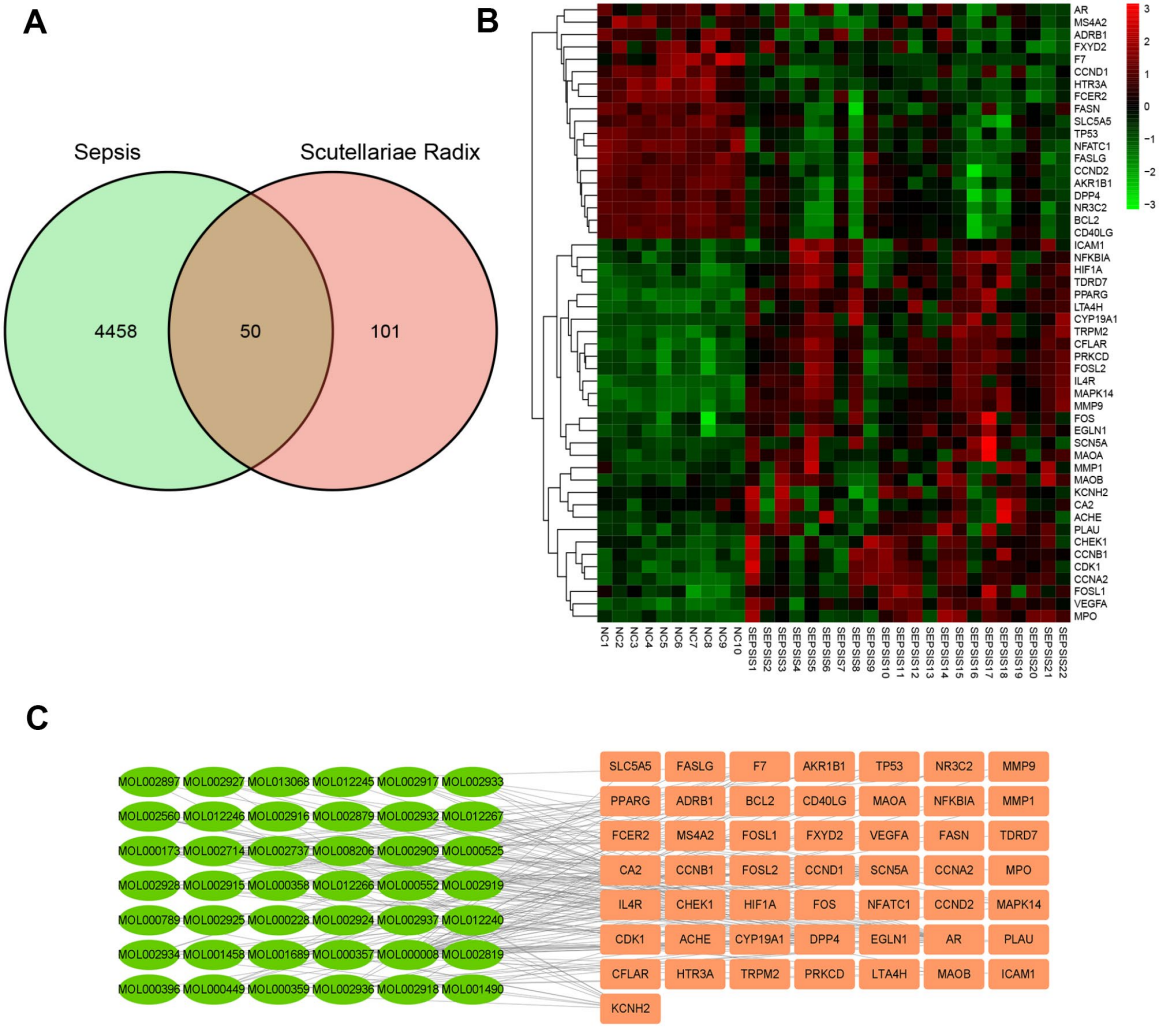
Target screening of scutellariae radix in the treatment of sepsis. (**A**), Venn diagram shows 4508 differentially expressed genes for sepsis in green, 151 active targets of Scutellariae Radix in pink, and 50 intersecting targets in the middle are defined as potential targets for the treatment of sepsis by Scutellariae Radix. (**B**), Heat maps based on Bulk RNA sequencing show that PRKCD, CFLAR, EGLN1, IL4R, etc. are highly expressed in sepsis, and AR, MS4A2, ADRB1, FXYD2, etc. are low in sepsis. (**C**), The ingredient-target network shows that the pink on the right represents 50 intersecting targets, the green ○ on the left represents the 42 active ingredients acting on the target

Using the TCMSP database, set DL ≥ 0.18, then obtained 72 Scutellariae Radix active ingredients. Following the correction of the official gene name abbreviations in the Uniprot database, 151 potential targets were obtained after removing non-human and non-standard targets. After the intersection with 4508 differentially expressed RNAs of sepsis patients, 50 intersection targets were obtained (Fig. 3A). The heat map based on Bulk RNA Sequencing data showed that PRKCD, CFLAR, EGLN1, IL4R, etc. were highly expressed in sepsis, AR, MS4A2, ADRB1, FXYD2, etc. were highly expressed in normal (Fig. 3B). The 42 active ingredients of Scutellariae Radix corresponded to 50 intersection targets (Fig. 3C).

### GO analysis

**Fig. 4:**
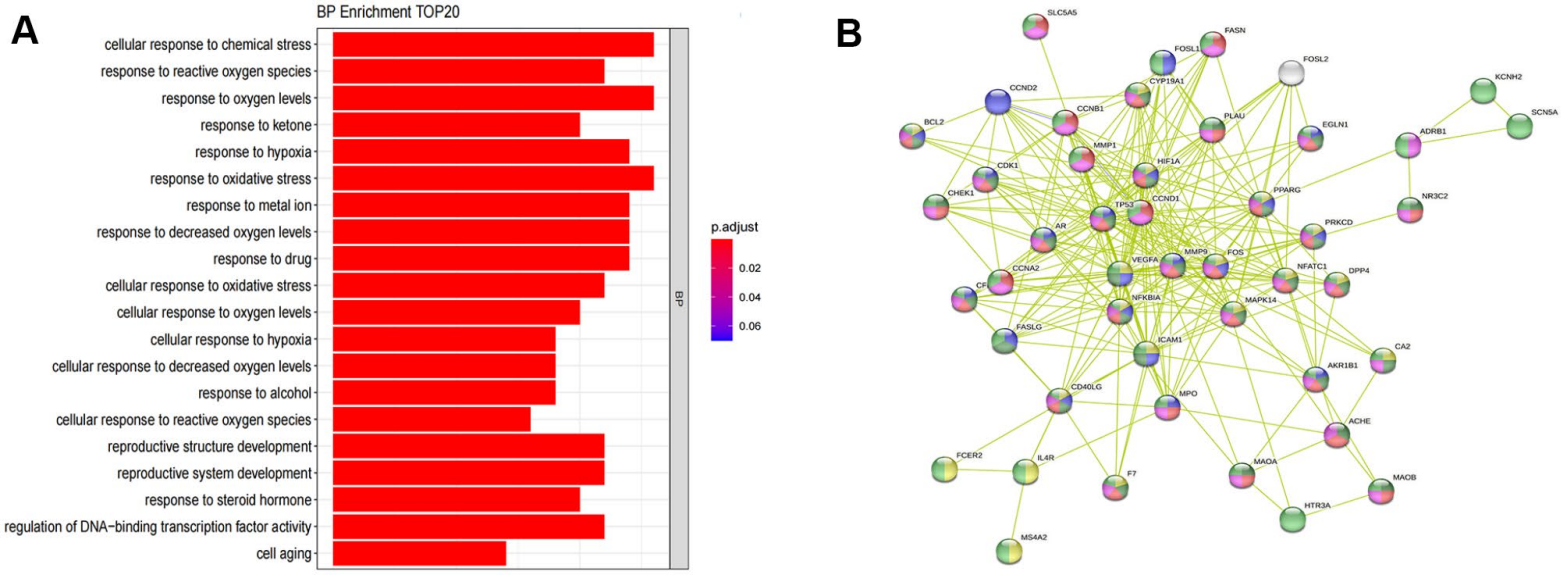
Intersection targets analysis. (**A**), BP results showed that intersection targets were mainly involved in cellular response to chemical stress, response to reactive oxygen species, response to oxygen levels, response to ketone, response to hypoxia, response to oxidative stress, response to metal ion, response to decreased oxygen levels, response to drug and other biological processes. (**B**), PPI results showed that 50 intersecting targets were closely connected, mainly involved in regulation of immune system process, regulation of cell death, regulation of cell communication, organic substance metabolic process, response to Stimulus and other biological processes

GO enrichment analysis showed that the intersection targets were mainly involved in cellular response to chemical stress, response to reactive oxygen species, response to oxygen levels, response to ketone, response to hypoxia, response to oxidative stress, response to metal ion, response to decreased oxygen levels, response to drug, cellular response to oxidative stress, cellular response to oxygen levels, cellular response to hypoxia, cellular response to decreased oxygen levels and other biological processes (Fig. 4A).

### PPI analysis

Among the 50 nodes and 257 connections of the PPI network, VEGFA, MMP9, TP53, CCND1, HIF1A, and other proteins were found in the middle (Fig. 4B), which may be potentially useful as targets for further research. Different colors represent different biological processes, among which, yellow represents regulation of immune system process, dark purple represents regulation of cell death, dark green represents regulation of cell communication, and red represents organic substance metabolic process, light purple represents metabolic process and light green represents response to stimulus.

### Survival analysis

**Fig. 5:**
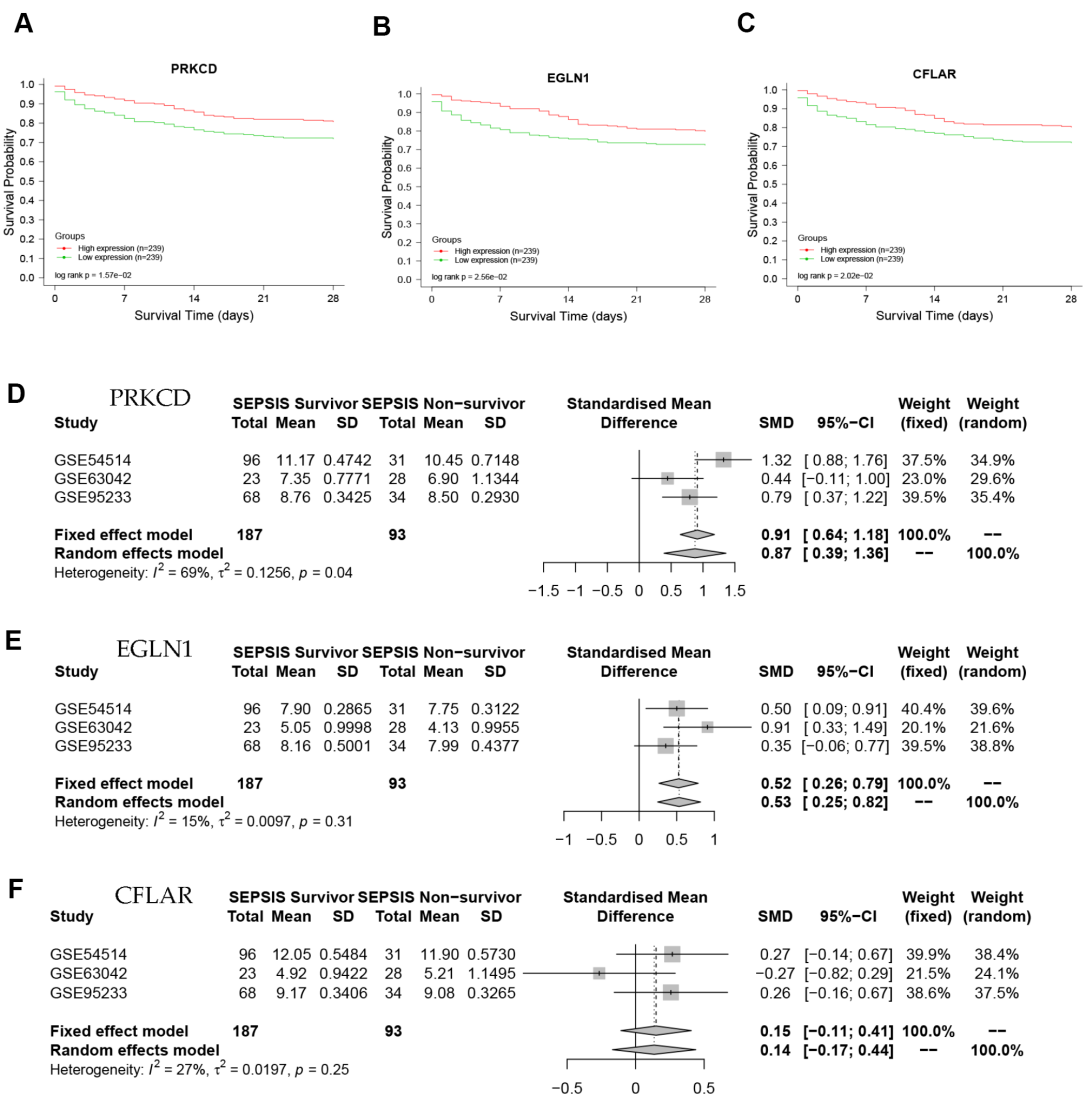
Survival curve and meta-analysis. (**A**-**C**), Based on the public dataset GSE65682, a total of 478 patients with sepsis were included in the analysis. In the survival curve, the abscissa represents the survival days, and the ordinate represents the survival rate. The red curve indicates the high expression group of the related genes, and the green curve indicates the correlation based on the low expression group. Statistical tests were performed using the logrank test. Survival curve showed the 28-day survival rate of septic patients, and it can be seen from the figure that the survival rates of patients with high expression of PRKCD, EGLN1 and CFLAR are higher than those in patients with low expression (*P* < 0.05). It means that high expression of PRKCD, EGLN1 and CFLAR is a protective factor for sepsis patients. (**D**-**F**), Based on the datasets GSE54514, GSE63042 and GSE95233, we performed a meta-analysis of the above three core genes in the normal and sepsis group. The data was tested for heterogeneity (I^2^), using a random effects model at I^2^>50%, and a fixed effects model at I^2^ ≤ 50%. For PRKCD, I^2^=69%, using a random-effects model, standardized mean difference (SMD) = 0.87, 95% confidence interval (CI): 0.39–1.36. For EGLN1, I^2^=15%, using a fixed effects model, SMD = 0.52, CI: 0.26–0.79. For CFLAR, I^2^ = 27%, using a fixed effects model, SMD = 0.15, CI: -0.11-0.41. It showed that PRKCD, EGLN1 and CFLAR were high expression in the sepsis survival group and low expression in non-survivor group

By plotting the survival curve, we found that the sepsis patients with high expression of Protein kinase C delta (PRKCD), Egg-laying defective nine 1 (EGLN1) and Fas-associating protein with a novel death domain (FADD)-like apoptosis regulator (CFLAR) had a higher 28-day survival rate than those with low expression of them(*P* < 0.05). This results suggested that these genes were related to the prognosis of septic patients, and their high expression may improve the prognosis of septic patients (Fig. 5A-C).

### Meta-analysis

The datasets GSE54514, GSE63042 and GSE95233 in GEO database were used to meta-analyze the above core genes at the transcription level, and the results showed that CFLAR, EGLN1 and PRKCD were highly expressed in the sepsis survivor group, lowly in non-survivor group (Fig. 5D-F). The correspondence between the three potential targets and the active ingredient of Scutellariae Radix is shown in Table 2.

**Table 2:** Active ingredients and targets of Scutellariae Radix

Ingredient	Mol-ID	MW	AlogP	HL	BBB	OB(%)	DL	Target
Wogonin	MOL000173	284.28	2.59	17.75	0.04	30.68	0.23	PRKCD
Baicalein	MOL002714	270.25	2.33	16.25	-0.05	33.52	0.21	EGLN1
Apigenin	MOL000008	270.25	2.33	-	-0.61	23.06	0.21	CFLAR

The three active ingredients Wogonin, Baicalein, Apigenin and their molecular ID (Mol-ID), molecular weight (MW), partition coefficient between octanol and water (AlogP), drug half-life (HL), blood-brain barrier (BBB), oral bioavailability (OB), drug-likeness (DL) and corresponding targets

### Single-cell RNA sequencing

**Fig. 6:**
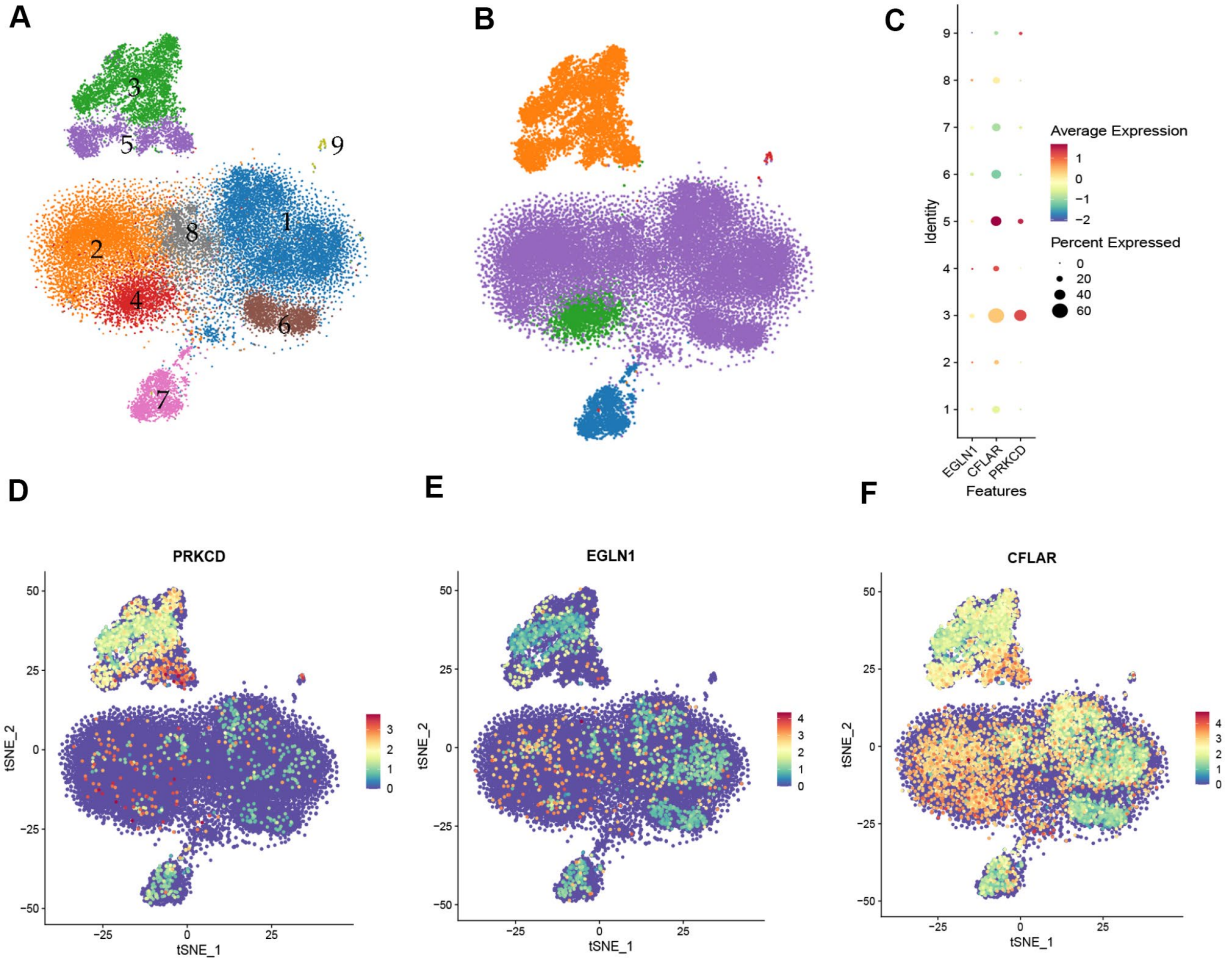
Single-cell RNA sequencing. (**A**), Mixed sample sequencing plot. The different colors represent cell lines containing different potential marker genes that are upregulated relative to the other cell lines. Where 1, 2, 6 and 8 represent T cells, 3 and 5 represent Macrophages, 4 represent NK cells, 7 represent B cells, and 9 represent Platelets. (**B**), Further visualization of the sequencing results of mixed samples. Different colors represent different cells. Orange represents Macrophages, purple represents T cells, green represents NK cells, blue represents B cells and red represents Platelets. (**C**). Each number in the ordinate represents a different cell line, with consistency with Fig. 6A. Different colors represent the average expression of the different cells. The diameter of the circle represents the percentage of the cells expressed. (**D**-**F**), Cell localization map showed that PRKCD were mainly localized in Macrophage cell lines, EGLN1 and CFLAR were widely localized in immune cells lines

After quality control, the number of high-quality cells for each sample was distributed in 3108 to 8509, the average number of genes in each cell was distributed in 343 to 2337, and after dimensionality reduction clustering, it was divided into 9 groups of cells (Fig. 6A), then the cell types identified by marker gene for reference were T cells, Macrophages, NK cells, B cells, Platelets (Fig. 6B). Among them, 1, 2, 6 and 8 represent T cells, 3 and 5 represent Macrophages, 4 represent NK cells, 7 represent B cells, and 9 represent Platelets. According to single-cell RNA sequencing results, PRKCD is mainly localized in Macrophage cell lines, EGLN1 and CFLAR are widely localized in immune cell lines (Fig. 6C-F).

### Molecular docking

**Fig. 7:**
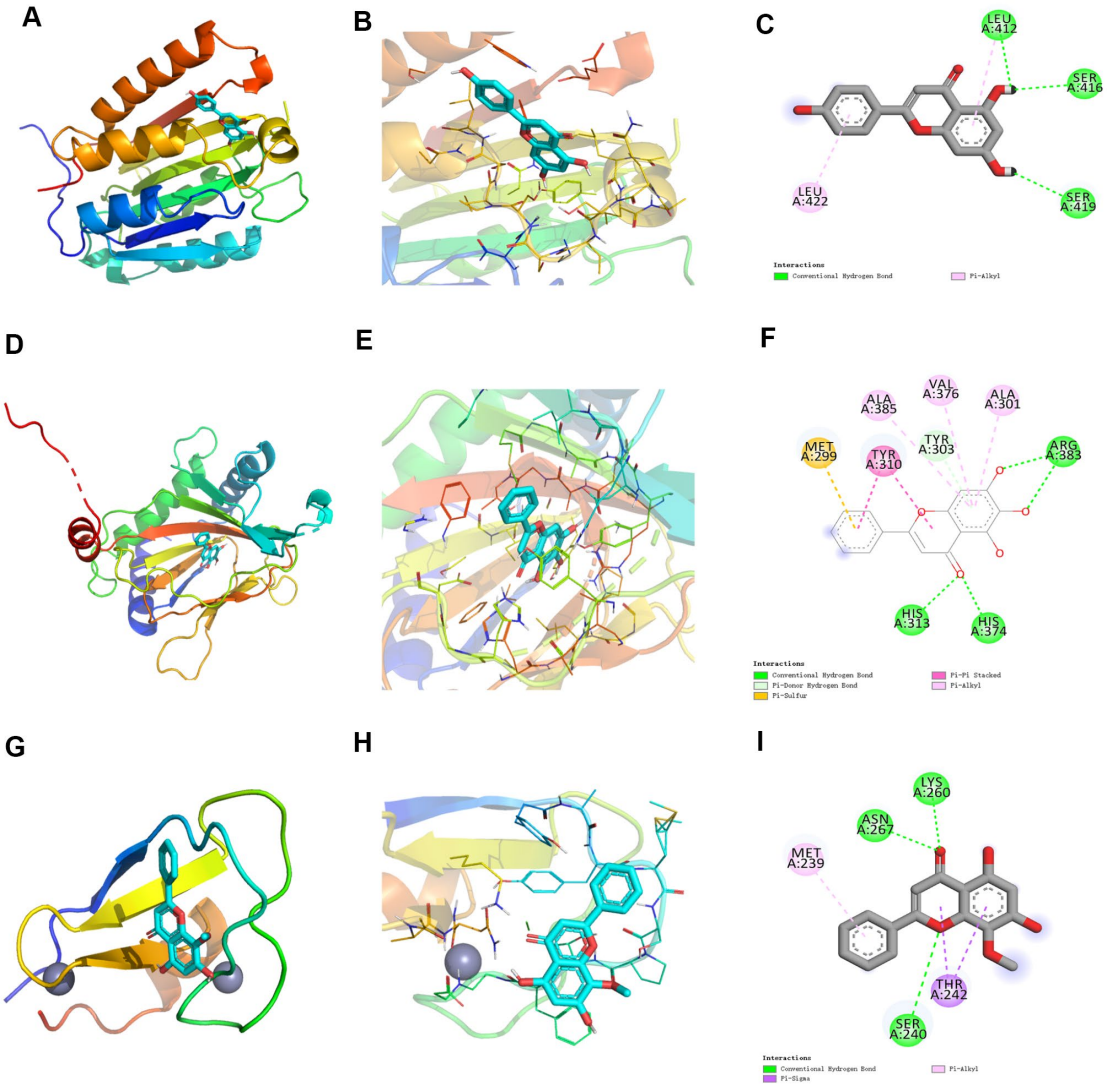
Molecular docking. (**A**-**C**), Binding mode of CFLAR and Apigenin based on molecular docking. CFLAR forms conventional hydrogen bond interactions with LEU412, SER416 of Apigenin, carbon-hydrogen bond interactions with SER419, and Pi-Alkyl interactions with LEU422. Molecular docking results showed that the Affinity values of CFLAR and Apigenin was − 6.7 kcal/mol. (**D**-**F**), Binding mode of EGLN1 and Baicalein based on molecular docking. EGLN1 forms conventional hydrogen bond interactions with HIS313, HIS374, ARG383 of Baicalein, Pi-Donor Hydrogen Bond interaction with TYR303, Pi-Pi Stacked interaction with TYR310, and Pi-Alkyl interactions with ALA301, VAL376, ALA385. Molecular docking results showed that the Affinity values of EGLN1 and EGLN1 was − 8.1 kcal/mol. (**G**-**I**), Binding mode of PRKCD and Wogonin based on molecular docking. PRKCD forms conventional hydrogen bond interactions with SER240, LYS260, and ASN267 of Wogonin, a Pi-Sigma interaction with THR242, and a Pi-Alkyl interaction with MET239. Molecular docking results showed that the Affinity values of PRKCD and Wogonin was − 6.0 kcal/mol

The target protein is preprocessed to make it a low-energy conformation that satisfies the ligand structure. The Affinity (unit: kcal/mol) value represents the binding capacity of the two, and the lower the binding energy, the better the binding activity, and the more stable the binding between the ligand and the receptor. It is generally believed that the affinity value less than − 4.25 kcal/mol indicates some binding activity between the two, less than − 5.0 kcal/mol indicates good binding activity, and less than − 7.0 kcal/mol indicates strong binding activity. The molecular docking results are shown that the affinity value of Apigenin (4’,5,7,-trihydroxyflavone) with CFLAR was − 6.7 kcal/mol, the affinity value of Baicalein (5,6,7-trihydroxy-2-phenyl-4 H-1-benzopyran-4-one) with EGLN1 was − 8.1 kcal/mol, and the affinity value of Wogonin (5,7-dihydroxy-8-methoxyflavone) with PRKCD was − 6.0 kcal/mol (Fig. 7A-I).

## Discussion

It has been widely accepted that uncontrolled systemic inflammatory responses and immune dysregulation are important in the pathogenesis of sepsis. Lipopolysaccharide (LPS) is a major component of the cell wall of Gram-negative bacteria, and the entry of LPS into the lymphatic and circulatory systems may cause a systemic inflammatory response. LPS, by activating immune cells such as macrophages and neutrophile granulocytes, initiates the innate immune system to resist inflammation, resulting in an increased secretion of a large number of proinflammatory cytokines, including tumor necrosis factor-α (TNF-α), IL-1, IL-6, NO, prostaglandin E2, and macrophage chemoattractant protein-1 (MCP-1) [2, 3]. The persistently produced proinflammatory cytokines can cause severe tissue destruction, and in addition, LPS activates Toll-like receptors, triggering a sepsis cascade. This cumulative inflammatory response ultimately leads to MODS, which is associated with high mortality in septic patients [22]. Clinically, the disease is characterized by cardiovascular, hepatic, pulmonary, renal, and gastrointestinal dysfunction. Treatment of sepsis with TCMs is characterized by multi-component, multi-target, multi-pathway, systematic regulation, and synergistic effects [5]. In this study, network pharmacology and combined RNA sequencing technology were used to select the active components of Scutellariae Radix, including Apigenin, Baicalein and Wogonin. All three are flavonoid compounds, which can produce anti-sepsis effects by regulating the expression of CFLAR, EGLN 1 and PRKCD, respectively, thus improving the survival rate of sepsis patients.

Apigenin is a non-toxic dietary flavonoid with biological activities such as anti-inflammatory, antioxidant and anticancer. Diffuse endothelial injury caused by sepsis leads to alveolar capillary injury, resulting in increased alveolar capillary membrane thickness and diffusion disorders, which causing ARDS or ALI. Cardenas et al. found that Apigenin inhibited neutrophil infiltration and apoptosis by regulating nuclear factor kappa B (NF-κB) signaling pathway to improve the inflammatory “cytokine storm” in lungs [23]. In addition, Cicek et al. found that Apigenin ameliorated lung injury by inhibiting the active caspase-3 pathway and proapoptotic Bax protein, thereby reducing the activation of oxidative stress response, resulting in suppressed pro-inflammatory cytokines and increased anti-inflammatory cytokines [24]. Cardiac dysfunction often accompanies sepsis patients, and through the inactivation of p38 mitogen-activated protein kinase, Apigenin has been shown to reduce myocardial ischemia-reperfusion injury [25]. Zhang et al. found that serum CK-MB, LDH, TNF-α, IL-6, and IL-1β levels were significantly reduced in the septic mouse model after Apigenin treatment, possibly through the inhibition of sphingosine kinase 1/sphingosine 1-phosphate signaling pathway [26]. The inhibition of NF-κB signaling pathway and the activation of autophagic pathway regulator TFEB by Apigenin may also be one of the mechanisms of reduce inflammatory factors and oxidative stress in sepsis [27]. In addition, the apoptosis of endothelial cells is promoted by LPS, which increases the production of reactive oxygen species (ROS), induces mitochondrial dysfunction and alters metabolic function, which also contributes to myocardial injury in sepsis. According to Silvia et al., Apigenin directly targets isocitrate dehydrogenase 3 (IDH 3), an enzyme involved in tricarboxylic acid cycle [28]. Apigenin can reduce ROS production and restore mitochondrial function by regulating the activity of IDH 3 and respiratory chain Complex I. Meanwhile, by inhibiting caspase-3, a cell apoptosis regulator, apigenin reduced endothelial cell apoptosis in response to LPS. As a result of these mechanisms, inflammatory factors and oxidative stress are suppressed, metabolic disorders are ameliorated, and sepsis-induced cardiac damage is protected against. Liver dysfunction is an early sign of sepsis and an independent risk factor for the poor outcome of sepsis. LPS causes endothelial cell damage to induce the release of ROS and reactive nitrogen species (RNS), lipid peroxidation and destruction of lipid bilayer of cell membrane, which is an important cause of hepatocyte damage in sepsis. Mehmet et al. demonstrated that Apigenin can inhibit the signaling cascades of NF-κB p65 and MAPKs, reduce oxidative stress and neutrophil infiltration, and increase enzymatic and nonenzymatic antioxidant levels in liver cells, thus treating LPS-induced liver injury [29].

CFLAR is a negative regulator of cardiac remodeling and heart failure, involved in the regulation of biological processes such as apoptosis, inflammatory response and fibrosis in multiple organs [30]. A study by Wang et al. also found that CFLAR regulates cerebral ischemia-reperfusion injury and may exert neuroprotective effects by regulating inflammatory responses and endoplasmic reticulum stress [31]. In this study, we found that CFLAR was widely expressed in immune cells, mainly involved in cell death, cell communication, metabolic response, stimulus response and other processes. Among sepsis patients, the survival rate was higher in the high CFLAR expression group than in the low expression group. CFLAR is one of the core targets of Apigenin for sepsis. Meanwhile, the molecular docking results also suggested a good binding activity of Apigenin to CFLAR. Apigenin improves the survival rate of sepsis patients by regulating the expression of CFLAR, and the mechanism may be related to the inhibition of inflammatory response, the regulation of endothelial cell apoptosis, and the improvement of mitochondrial function.

Baicalein is the active ingredient of Scutellaria Radix, with antiviral, anti-inflammatory, anti-thrombosis, antioxidant and anti-liver fibrosis biological effects. Circulatory failure is one of the characteristics of advanced sepsis, and its mechanism may be related to the massive production of NO caused by LPS-dependent induced inducible NO synthase (iNOS). NO can react with superoxide anion to form the peroxynitrite anion, which oxidizes sulfhydryl groups and generates hydroxyl radical, and the production of these free radicals causes intractable hypotension and causes oxidative damage in the liver, lung and other organs [32]. Baicalein can inhibit the TNF-α and iNOS induced by LPS and reduce the generation of NO and free radicals, thus improving the shock symptoms in sepsis. In addition, myocardial inhibition is also one of the important causes of circulatory failure in sepsis. Lee et al. found that Baicalein could protect the myocardium by inducing heme oxygenase-1 and inhibiting ROS production. Meanwhile, the inhibition of MCP-1 by Baicalein also reduces the infiltration of macrophages in the myocardial tissue and reduces the apoptosis of cardiomyocytes [33]. Baicalein can also inhibit the activation of NF-κB and MAPKs signaling pathway, reduce the release of proinflammatory factors such as high mobility group box 1 (HMGB1) and macrophage migration inhibitory factor (MIF) by macrophages and neutrophils, and then reduce the apoptosis of hepatocytes [34]. Moreover, Baicalein can inhibit intestinal mucosal apoptosis by inhibiting pro-apoptotic gene Bax expression while up-regulating anti-apoptotic gene Bcl-2 expression, thus improving intestinal and liver damage in sepsis [35].

EGLN1 encodes the hypoxia-inducible factor (HIF) pathway prolyl hydroxylase 2 (PHD2) that serves as an oxygen-sensitive regulator of HIF activity [36]. HIF-1α is a regulator of cellular adaptation to hypoxia, which can be hydroxylated by EGLN1 in normoxia and subsequently degraded by proteases. Under hypoxia, HIF-1α can bind with HIF-1β to form heterodimers and exert effects on glycolytic metabolism, angiogenesis, cell proliferation and apoptosis. In this study, we found that sepsis patients with high EGLN1 expression had a better prognosis than those with EGLN1 low expression by multi-dimensional RNA sequencing. EGLN1 is an important target of Baicalein, and Baicalein can affect the expression of EGLN1 to produce anti-sepsis effect. Moreover, the molecular docking also indicates that they have a strong binding activity. The mechanism may involve the regulation of hypoxia in various tissues or organs, and the release of inflammatory substances such as TNF-α, MCP-1, HMGB1, MIF, and NO [37].

Wogonin is a common TCM for inflammation, allergy and tumors. The anti-inflammatory and antioxidant effects of Wogonin have been demonstrated in many diseases. Specifically, Wogonin suppresses the oxidative stress response to treat non-alcoholic steatohepatitis by activating peroxisome proliferator-activated receptor-γ (PPAR-γ) and adiponectin receptor 2 (AdipoR2) pathway [38], and it can also reduce nephrotoxicity by regulating PPAR-γ and NF-κB signalling to produce antioxidant effects [39]. Meanwhile, Wogonin attenuates septic hepatic damage by inhibiting Nrf2-mediated NF-κB signalling [40]. Furthermore, in the study of Soyoung et al., Wogonin can inhibit the expression of endothelial cell adhesion molecules (CAMs), as well as affect the migration and aggregation of monocytes to Primary human umbilical vein endothelial cells (HUVECs), thus reducing HMGB1 release and mucosal barrier disruption caused by LPS and cecal ligation and puncture (CLP) [41].

PRKCD belongs to the the PKC family and is an important regulator of neutrophil-endothelial cell interactions [42]. In neutrophils, PRKCD can regulate NF-κB signalling and affect the production of ROS as well as the secretion of cytokines such as TNF-α, IL-1. In endothelial cells, PRKCD is involved in NF-κB signalling regulation, adhesion molecule expression, the release of inflammatory mediators and other biological processes. Previous studies have found that PRKCD is an important regulator of mitochondrial dysfunction, and is associated with myocardial contractility during sepsis [43]. In this study, single-Cell RNA sequencing found that PRKCD was mainly localized in macrophage cell lines, which was highly expressed in the septic survival group, and was positively correlated with the prognosis of septic patients. However, this result seems to be contrary to the conclusions of previous studies on animal models of sepsis [44], which may be caused by individual differences in septic animal models and sequenced samples, or the different stages of anti-inflammatory/immunosuppression in septic patients. PRKCD is one of the targets of Wogonin, and they have a good binding activity. PRKCD may participate in the development of sepsis by regulating the inflammatory response in neutrophil-endothelial cells, and the mitochondrial function of cardiomyocytes. However, the specific mechanism of action still requires further studies in animal models and clinical studies in large samples.

In conclusion, this study, using network pharmacology combined with RNA sequencing, revealed that Apigenin, Baicalein and Wogonin could improve the poor prognosis and improved survival of sepsis patients by separately regulating the expression of their targets CFLAR, EGLN1 and PRKCD. This study can help us to further understand the relationship between the active ingredients of Scutellaria Radix, targets and sepsis. Furthermore, the possible action targets and lead compounds of new drug for treating sepsis may be developed based on results from this study.

## Conclusions

Apigenin, Baicalein and Wogonin, important active components of Scutellaria Radix, produce anti-sepsis effects by regulating the expression of their targets CFLAR, EGLN1 and PRKCD. These findings provide us with new knowledge about the role of Scutellaria Radix in sepsis and bring new approaches for the treatment of sepsis.

## Data Availability

RNA sequencing datasets for this study are available in the China National GeneBank DataBase (CNGBdb) at https://db.cngb.org/. Accession codes: CNP0002611.
